# Simultaneous determination of lincomycin, spiramycin, tylosin and tiamulin in animal feed using liquid chromatography coupled to mass spectrometry after a microbial assay

**DOI:** 10.2478/jvetres-2025-0073

**Published:** 2025-12-23

**Authors:** Monika Przeniosło-Siwczyńska, Aleksandra Grelik, Maja Chyłek-Purchała

**Affiliations:** Department of Microbiological Research of Food and Feed, Department of Chemical Research of Food and Feed, National Veterinary Research Institute, 24-100 Puławy, Poland; Department of Chemical Research of Food and Feed, National Veterinary Research Institute, 24-100 Puławy, Poland

**Keywords:** antibiotics, antibiotic growth promoters, feed analysis, LC-MS, microbial assay

## Abstract

**Introduction:**

To monitor the occurrence of antibiotics in feed, a two-step control strategy is often adopted of screening by microbiological inhibition followed by confirmation by chromatographic techniques. This study is devoted to the development of a reliable method for simultaneous determination of lincomycin, spiramycin, tylosin and tiamulin in animal feed using liquid chromatography coupled to mass spectrometry after a microbial assay.

**Material and Methods:**

The analytes were extracted from feed using a methanol:water mixture, and solid-phase extraction was employed for the isolation of the antibiotics. The determination of the presence of lincomycin, spiramycin, tylosin and tiamulin was carried out using high-performance liquid chromatography with mass spectrometry.

**Results:**

The method was validated according to EU requirements. The decision limit and detection capability were 0.213–0.318 and 0.259–0.535 mg kg^−1^, respectively, and the limits of detection and quantification were 0.029–0.151 and 0.069–0.223 mg kg^−1^, respectively, depending on the analyte. Recoveries were satisfactory (86.6–105.1%), repeatability ranged from 2.6 to 18.3% and reproducibility from 6.2 to 11.7%.

**Conclusion:**

The proposed method is reliable and applicable to identify four antibiotics for feed-safety control.

## Introduction

Since the discovery and development of the first antibiotics, these medicines have played an important role in curing bacterial diseases in humans and animals. In modern agricultural practice, antibacterial agents are used on a large scale and administered to treat or prevent diseases, or to promote the growth of animals. The application of growth-promoting agents is strictly regulated, and the EU has prohibited antibiotics from being added to feed to stimulate the growth of animals since 1 January 2006 (7). However, therapeutic treatment with antibiotic drugs is often essential, as is prophylactic use of them to prevent the disease spreading.

Lincomycin (LIN) is a broad-spectrum lincosamide antibiotic that is derived from *Streptomyces lincolnensis* and shows activity against staphylococci and streptococci. Lincomycin is used in both human and veterinary medicine. In veterinary practice, it is widely used for the treatment of gastrointestinal and respiratory infections caused by lincomycin-sensitive microorganisms in a wide variety of food-producing animals such as pigs and poultry. In these animals, the drug is administered orally either by water medication or in-medicated feed. Spiramycin (SPI) and tylosin (TYL) belong to the class of 16-membered macrolide antibiotics. These are highly active against a wide range of Gram-positive bacteria, as well as against some Gram-negative bacteria, *Mycoplasma* and *Chlamydia*. Tylosin is used exclusively as a veterinary drug, and both it and spiramycin were approved in the EU as growth promoters (antibiotic growth promoters, AGPs) for production enhancement of food animals. The authorisation of these substances as feed additives was withdrawn in EU in 1999 (8) and has not been granted again. Currently tylosin is authorised as an active substance that can be used therapeutically in medicated feed. Tiamulin (TIA) is a semisynthetic derivative of the naturally occurring antibiotic pleuromutilin produced by the fungus *Pleurotus mutilus*. It is a limited-spectrum antibiotic, highly active against staphylococci, streptococci and mycoplasmas. Tiamulin is used exclusively in veterinary medicine and is widely prescribed because of its good properties. Tiamulin is suitable for administration to animals in medicated feed or drinking water, or by injection.

Whilst the in-feed administration of veterinary medicines is an essential treatment/prophylaxis route for intensively reared species, contamination of feed by carryover can and does occur (16). Deleterious effects may occur in both the animals ingesting the contaminated material and the people consuming products from these animals. Feed contamination can produce toxicological effects in animals (*e.g*. tiamulin and ionophore toxicity from their interaction). Transmission of antibiotics through the food chain to food products of animal origin containing their residues and finally to humans can result in different adverse effects, the development of drug resistance in intestinal bacteria populations, immune suppression, allergic reactions, hepatic and renal dysfunctions, and other issues. Antibacterial agents may enter the food chain as a result of illegal, injudicious or incorrect use of antibiotics or through cross-contamination of feed related to manufacturing of medicated feed (10, 16).

To monitor the veterinary drug residues in food of animal origin, a two-step control strategy is often adopted: screening by microbiological inhibition followed by confirmation by chromatographic techniques. A similar approach is proposed for official monitoring of banned antibiotics and growth promoters in feedingstuffs (5). Microbiological methods, which play an important role among screening methods, are usually based on inhibition of the growth of test strains in a specific agar medium by the antibacterial substances contained in the sample. They have found wide application in residue analysis of antibiotics in animal tissues and products. As agar diffusion methods, they have also been employed in the analysis of feed (5, 12, 19). Microbiological methods have the capability for high sample throughput and are used to screen large numbers of samples. In recent years there has been a rapid development of methods for the analysis mainly of food matrices for veterinary antimicrobial drugs. Although there was also a need for analytical methods to monitor the use of veterinary drugs in feed, only a limited number of them were developed and made available.

The main purpose of this study was to develop a simple and reliable method for the simultaneous determination of lincomycin, spiramycin, tylosin and tiamulin using high-performance liquid chromatographic (HPLC) separation followed by mass spectrometric detection and demonstrating competence in three antimicrobial classes: lincosamides, macrolides and pleuromutilins. Our previous work was focused on the development of the method for the determination of tylosin (20). Where an adequate multi-analyte method was needed which could be implemented in an overall control strategy, the presented method for confirmation of positive results first obtained in microbiological multi-screening was developed. To the best of the authors’ knowledge, no method has yet been published combining four antibiotics belonging to three groups, namely macrolides (tylosin and spiramycin), lincosamides (lincomycin) and pleuromutilins (tiamulin). Most chromatographic methods developed by other researchers are not able to detect these four substances as confirmation of screening results.

## Material and Methods

### Chemicals and reagents

Lincomycin, spiramycin, tylosin, tiamulin and formic acid were purchased from Sigma-Aldrich (St. Louis, MO, USA). Methanol was obtained from Avantor Performance Materials (Gliwice, Poland) and HPLC-grade acetonitrile and methanol were purchased from J.T. Baker (Deventer, the Netherlands). Water was purified with the Milli-Q water purification system from Millipore (Bedford, MA, USA). Oasis HLB solid-phase extraction cartridges (60 mg, 3 mL) were purchased from Waters (Milford, MA, USA).

### Standard solutions

To prepare stock standard solutions of individual compounds, 10 ± 0.01 mg of each analyte was weighed into 10 mL volumetric flasks and dissolved in methanol to yield a final concentration of 1 mg mL^−1^. The standard solutions were stored in the dark at –18°C for no longer than three months. Working standard solutions of 0.1 mg mL^−1^ were freshly prepared by dilution of the stock solutions with methanol.

### Preparation of matrix-based standard solutions

Working standard solutions of lincomycin, spiramycin, tylosin and tiamulin were prepared in HPLC-grade methanol at a concentration of 0.1 mg mL^−1^ to prepare a set of matrix-based standards with concentrations ranging from 0.25 to 2 mg kg^−1^. Matrix-based standards were prepared by extracting 5 g of blank feed fortified with working standard solutions according to the procedure presented in the following section.

### Sample collection

Feed samples were collected by veterinary inspectors within the confines of the national feed control plan in 2020–2024. The samples represented various types of feed, mainly for pigs and poultry. A total of 342 feed samples were analysed to detect antibacterial substances. In the first stage, the samples were screened using a microbial assay according to the feed control strategy adopted in Poland (19). Out of these 342 samples, 31 were found to be positive and suspected to have macrolide, lincosamide or pleuromutillin present.

### Microbial assay

A sample was considered positive if a growth inhibition zone was created around it on an agar test plate (pH 8.0) inoculated with *Kocuria rhizophila* ATCC 9341 and designed to detect macrolides (SPI and TYL in this case), lincosamides (LIN) and pleuromutilins (TIA). All samples showing growth inhibition zones on this test plate were further analysed and confirmed using liquid chromatography coupled to mass spectrometry (LC-MS).

### Sample extraction and clean-up

Feed samples, including negative and positive control samples were homogenised and 5-g masses of them were weighed into volumetric flasks. Then, 20 mL of extraction mixture (methanol/water (50/50, v/v) was added to each. The samples were shaken on a horizontal shaker for 30 min and centrifuged for 10 min (4,000 x *g*) and then 3 mL of the supernatants were diluted with 7 mL of water. Then the total volumes of extracts were purified by solid phase extraction (SPE) using a 12-port Baker vacuum system and Oasis HLB cartridges, which were preconditioned with 3 mL of methanol followed by 5 mL of water. The cartridges were washed with 3 mL of water and dried under vacuum for one min, and then the analytes were eluted with 2 mL of methanol. After evaporation in a nitrogen stream, the residues were reconstituted in 2 mL of the aqueous mobile phase, passed through a nylon membrane filter (0.45 μm) and subjected to the chromatographic analysis.

### LC-MS analysis

The LC-MS system consisted of an Agilent 1200 series liquid chromatograph equipped with a binary pump, a degasser, an autosampler, a column heater and a single quadrupole mass analyser (MSD 6140 Agilent). Compound separation was achieved using Zorbax Eclipse XDB-C18 column 150 mm x 4.6 mm, 5 μm (Agilent, USA). The mobile phase, a mixture of 0.05 M formic acid in water as solvent A and 0.05 M formic acid in acetonitrile as solvent B, was delivered to the analytical column according to the gradient programme shown in Table 1. The flow rate was 0.3 mL min^−1^, the sample injection volume was 10 μL, the column temperature was maintained at 35°C and the run time was 35 min. Electrospray ionisation (ESI) was set in the positive mode. The fragmentor voltage was set at 100 V for all monitored antibiotics, the drying gas flow was 11.0 L/min and the temperature was 300°C. The capillary voltage was set at 3,000 V and the nebuliser pressure was 35 psi. Selected ion monitoring was used to detect the analysed antibiotics, and the protonated molecular (M+H)+ ions (m/z) monitored were those indicated in Table 2. The analytes were identified by the retention time and the protonated molecular ion (M+H)+ and quantification was based on the calibration curves prepared by spiking blank feed samples and constructed by plotting the peak area *versus* the antibiotic concentrations.

**Table 1.** The gradient programme followed for the elution of the examined analytes

t (min)	A: 0.05M CH_2_O_2_ in water (%)	B: 0.05M CH_2_O_2_ in acetonitrile (%)
0	95	5
3	73	27
11	73	27
13	50	50
20	50	50
22	95	5
35	95	5

**Table 2.** Monitored ion (m/z) and retention time of analysed antibiotics

Antibiotic	Monitored ion (m/z)	Retention time (min)
Lincomycin	407	10–11
Spiramycin	422	11–12
Tylosin	916.5	20–21
Tiamulin	494	21–22

### Method validation

Linearity, precision (repeatability and intra-laboratory reproducibility), recovery, decision limit (CCα), detection capability (CCβ), limit of detection (LOD), limit of quantification (LOQ) and selectivity were analysed according to the criteria of Commission Decision 2002/657/EC in force at the time (9). Blank feed samples were spiked with the analysed compounds at 0.25, 0.5, 1.0, 1.5 and 2.0 mg kg^−1^ for linearity verification. To evaluate precision, blank feed samples were spiked at 0.25, 1.0 and 2.0 mg kg^−1^. Six replicates of each concentration were analysed on the same day to demonstrate repeatability. The procedure was repeated to determine within-laboratory reproducibility, as well as recovery, by comparing the results from samples prepared and analysed during three consecutive days. The CCα and CCβ values were calculated using within-laboratory reproducibility results. Moreover, LODs and LOQs values were estimated for the four compounds. Each compound’s LOD and LOQ value was calculated on the basis of signal-to-noise ratios and were S/N = 3 for LOD and S/N = 10 for LOQ. The selectivity of the method was investigated by the analysis of 20 blank feed samples (n = 20) to verify the absence of potential interfering compounds at the target analyte retention times.

### Application of the method

The presented method was evaluated in proficiency tests for antibiotics in animal feed organised by Wageningen Food Safety Research in the Netherlands. Our laboratory reported correct results without any false positives or negatives and the *z*-scores were satisfactory.

The developed method was also applied to real feed samples. The multi-analyte method was adopted for the confirmatory analysis of feed samples found positive by the microbiological method. The method was used to investigated 31 samples of feeds suspected of containing antibiotics and to determine the presence of lincomycin, spiramycin, tylosin or tiamulin in them.

## Results

### Method validation

Good linearity was obtained for the four selected compounds with correlation coefficients R^2^ ≥ 0.997 for all cases of matrix-matched calibration curves.

Adequate repeatability expressed as coefficients of variation (CV) was achieved with CVs between 2.57% and 18.33%, while CVs for reproducibility were in the range of 6.22% to 11.69%, depending on analyte and fortification level. Recovery of the analysed compounds varied from 86.60 % to 105.07%. The CCα and CCβ values were determined in the range from 0.213 to 0.318 mg kg^−1^ and from 0.259 to 0.535 mg kg^−1^, respectively. The values of the LOD fell between 0.029 and 0.151 mg kg^−1^, and those of the LOQ were from 0.069 mg kg^−1^ to 0.223 mg kg^−1^. Moreover, the method also proved to be selective because it produced no interfering peaks in the retention times of the analysed antibiotics. The validation data of the method are given in Tables 3 and 4.

**Table 3.** Validation parameters of the developed method for detection and quantification of four antibiotics in animal feed - linearity, decision limit (CCα), detection capability (CCβ), limit of detection (LOD) and limit of quantification (LOQ)

Target compound	Linearity R^2^	CCα mg kg^−1^	CCβ mg kg^−1^	LOD mg kg^−1^	LOQ mg kg^−1^
Lincomycin	0.9977	0.318	0.535	0.151	0.223
Spiramycin	0.9986	0.242	0.286	0.103	0.107
Tylosin	0.9989	0.294	0.317	0.029	0.069
Tiamulin	0.9970	0.213	0.259	0.065	0.147

**Table 4.** Validation parameters of the developed method for detection and quantification of four antibiotics in animal feed - repeatability, within-laboratory reproducibility, recovery and uncertainty

Target compound	Fortification level (mg kg^−1^)	Repeatability, CV (%)	Reproducibility, CV (%)	Recovery (%)	Uncertainty (%)
Lincomycin	0.25	18.33	11.62	91.23	23.4
	1.0	9.44	8.76	89.60	
	2.0	6.92	11.69	90.31	
Spiramycin	0.25	4.73	11.20	96.29	22.4
	1.0	5.66	7.84	101.05	
	2.0	3.80	7.18	97.51	
Tylosin	0.25	6.47	6.98	86.60	16.9
	1.0	4.30	7.04	104.44	
	2.0	8.26	8.45	96.63	
Tiamulin	0.25	7.62	7.29	102.81	14.6
	1.0	2.57	6.25	105.07	
	2.0	2.93	6.22	100.27	

### Results for real feed samples

The proposed method was used to determine the presence of lincomycin, spiramycin, tylosin and tiamulin in feed. Among 31 feed samples analysed for the antibiotics, 10 (32.3%) samples were found to be positive, 9 contained tylosin and 1 tiamulin. Tylosin was identified at concentrations ranging from 0.5 mg kg^−1^ to 63 mg kg^−1^. The concentration for tiamulin was determined at 67 mg kg^−1^.

## Discussion

Contamination of animal feed with undeclared antibiotics is an important problem for food safety and human health. Antibiotics in feed (including AGPs, but also medicated feed) have been the subject of much debate over recent years. It is believed that excessive use of antibiotics in animal production is one factor contributing to the global rise in antimicrobial resistance. That is why it is also important to ensure that antibacterial agents in animal production are monitored by the relevant authorities and that feed is investigated for the presence of antibiotics. The effective control of the possible illegal use of antibacterial substances requires the availability of multi-screening and confirmatory methods.

The microbiological assays perform very well as qualitative methods of screening for antibacterial substances. However, they tend to lack specificity, and for this reason are unsuitable for the identification of antibacterial substances. According to literature data, liquid chromatography was used to reveal individual antibacterial substances in feed combined with various types of detectors: UV (3, 6), FLD (2, 18), DAD (5, 29), MS (1, 4, 17, 25, 27, 28, 30). Liquid chromatography with diode-array detection and with mass spectrometry have proved to be effective techniques for the confirmation of screening-positive feed samples (5).

The main goal of the presented study was to develop a sensitive, specific and reliable multi-analyte method for confirmatory analysis when feed samples gave positive results in initial screening. One of the main advantages of the presented method is the possibility of simultaneous determination of lincomycin, spiramycin, tylosin and tiamulin in correlation with the microbiological method. The optimisation study was performed by identifying some factors, *i.e*. extraction, SPE clean-up, analytical separation and MS detection. Animal feeds are considered difficult matrices because of their variable and complex composition; therefore, the most appropriate extraction solvent and a good purification strategy are crucial protocol design criteria. In our study particular attention was paid to the optimisation of the sample preparation procedure, on account of the complexity of feed matrices. Several solvents were tested for the extraction of the antibiotics: water, methanol and acetonitrile in various ratios, all with and without the addition of formic acid. The extraction mixture of methanol and water (1:1) gave the best extraction recovery. Extraction using methanol and water was also the choice of other authors (3, 23, 26). Our method involved feed-sample extraction followed by a SPE-purification step which eliminated the major chromatographic interferences. Solid phase extraction was the most commonly used technique of feed sample purification (3, 11, 14, 17, 18, 23, 25, 26). In our study Oasis HLB sorbent successfully purified all analytes with high efficiency. The elution of the analytes was tested using mixtures of acetonitrile/water and methanol/acetonitrile and using pure methanol, resulting in 2 mL of methanol proving optimal for elution. Chromatographic conditions were optimised to improve separation, sensitivity and selectivity. From the literature, we knew that in general all the selected compounds could be chromatographed on C18 stationary phases (1, 3, 4, 18, 25, 26). The best results were achieved using a Zorbax Eclipse XDB-C18 column in combination with a mobile phase comprised of 0.05 M formic acid in water (eluent A) and 0.05 M formic acid in acetonitrile (eluent B). It was considered necessary to use gradient elution, and the addition of formic acid enhanced the peak resolution and sensitivity. Optimisation work was also undertaken on the mass spectrometry step. Positive ion mode was used for all analytes, and electrospray ionisation proved to yield the good results for them all.

The linearities were good for all analytes in the whole range of tested concentrations, as proved by the correlation coefficients greater than 0.997 for all curves. The obtained values of CCα and CCβ were highly satisfactory, as were the LOD and LOQ values. The recoveries of LIN, SPI, TYL and TIA were in the ranges of 89.6–91.2%, 96.3–101.1%, 86.6–104.4% and 100.3–105.1%, respectively; and were similar to those obtained and reported by other authors, where they variously ranged from 67 to 98% (26), from 88 to 102% (1), from 92 to 102% (21) and from 99.5 to 100.9% (30). The CVs obtained for the developed multi-analyte method were in the range of 2.6–18.3% for repeatability (CV_r_) and 6.2–11.7% for intra-laboratory reproducibility (CV_R_) and were comparable to those obtained by other authors: CV_r_ < 17% (25); 17% (CV_r_) and 22% (CV_R_) (4); CV_r_ < 8% and CV_R_ < 9% (1).

The main advantage of the method is its ability to detecting four antibiotics in one analytical procedure in combination with screening by microbiological method. The method allows the efficient and effective surveillance of the possible illegal use of antibiotics and growth promoters in feed either as banned AGPs (as spiramycin or tylosin) or as cross-contaminants after the production of medicated feed (in this case as lincomycin, tiamulin or tylosin). Most methods developed by other researchers did not offer the possibility of detecting these four antibiotics in one assay as confirmation of a screening test result.

The obtained results for real samples revealed that 10 (32.3%) of them contained undeclared antibiotics. Nine samples contained tylosin with concentrations ranging between 0.5 and 63 mg kg^−1^. These concentrations of tylosin could occur through intentional non-medicinal use of the antibiotic (for prophylaxis, growth promotion or arbitrary use by animal breeders). However, the explanation for the relatively low levels of the antibiotic could be cross-contamination, which may permeate a batch of feed after the manufacture of batches of medicated feed in a feed mill, may take place during feed transport to farms, or may even arise on the farm itself (in storage, manipulation and mixing operations, if medicated feed is provided). It should be noted that almost all positive samples contained tylosin, which may be related to the high production of feed medicated with this antibiotic in Poland. In three samples the determined concentrations of tylosin (41 mg kg^−1^ and 63 mg kg^−1^) and tiamulin (67 mg kg^−1^) implied that the antibiotics may have been used intentionally and illegally. The amounts were very high, and too high for cross-contamination, but on the other hand, too low for therapeutic doses, particularly because there was no declaration with the samples that they were medicated feed.

Comparison of our results with data from other countries is difficult due to the lack of detailed data on the analysed feeds and the range of analytes. Even within the EU, monitoring of the presence of antibacterial substances in feed varies across Member States. A study conducted in Northern Ireland revealed that 24.8% of the feed samples contained antimicrobial additives (15). The most frequently occurring substance was chlortetracycline (in 15.2% of samples), followed by sulphonamides, penicillin and ionophores. Studies on the presence of β-lactam antibiotics and sulphonamides in cattle feed revealed that of 21 samples, 18 (85.7%) contained these antibacterial substances (13). Other studies (22) found contamination of pig feed with tiamulin, previously used to produce medicated feed, at concentrations lower than 5 mg/kg (the LOQ of the method). Research conducted in Belgium showed that 46% of the feed samples contained residues of active substances previously used to produce medicated feed, 20% of which exceeded the acceptable contamination level of 1% of the therapeutic dose (21). A study conducted in the Netherlands (24) showed that a portion of flushing-feed samples collected after production of medicated feed was contaminated with antibiotic residues. Overall, contaminating antimicrobials were detected in 87.1% of all samples tested. The most frequently detected antibiotic was oxytetracycline; however, tylosin was detected in seven samples (5.7% of positive samples) with concentrations ranging between 0.6 and 6.0 mg kg^−1^.

## Conclusion

A new multi-analyte method for the identification and quantification of lincomycin, spiramycin, tylosin and tiamulin based on LC-MS was introduced. The proposed method provided good sensitivity, accuracy and precision for target compounds and was successfully applied to real feed samples. The sensitivity of the method is sufficient for the routine monitoring of antibiotics in animal feed and its satisfactory statistical parameters qualify it for application in routine laboratory assays.
